# Adsorption Study of Continuous Heavy Metal Ions (Pb^2+^, Cd^2+^, Ni^2+^) Removal Using Cocoa (*Theobroma cacao* L.) Pod Husks

**DOI:** 10.3390/ma15196937

**Published:** 2022-10-06

**Authors:** Candelaria Tejada-Tovar, Angel Villabona-Ortíz, Ángel González-Delgado

**Affiliations:** 1 Chemical Engineering Department, Process Design and Biomass Utilization Research Group (IDAB), Universidad de Cartagena, Avenida del Consulado St. 30, Cartagena de Indias 130015, Colombia; 2 Chemical Engineering Department, Nanomaterials and Computer Aided Process Engineering Research Group (NIPAC), Universidad de Cartagena, Avenida del Consulado St. 30, Cartagena de Indias 130015, Colombia

**Keywords:** adsorption, metals, biomass, cocoa

## Abstract

The serious toxicological effects of heavy metal ions in aquatic ecosystems have motivated the search for alternatives to reduce contamination of water sources from industrial wastewater. In this work, continuous adsorption of nickel, cadmium, and lead was assessed using a packed bed column filled with Cocoa (*Theobroma cacao* L.) pod husks widely available in the northern region of Colombia. The physicochemical characterization of the agricultural biomass was performed to quantify its chemical composition by bromatological, FT-IR, and energy-dispersive X-ray spectroscopy (EDS). The breakthrough curves were constructed for all heavy metal ions with bed depth of 4 and 7.5 cm, taking aliquots at 10, 30, 60, 90, 120, 150, 180, 210, 240, and 270 min. Moreover, experimental data were fitted to adsorption models in continuous mode to predict adsorptive performance (Adams–Bohart, Thomas, and Yoon–Nelson). For the FT-IR analysis of biomass before and after adsorption, the most representative bands occur around 3200–3900 cm^−1^ attributed to the presence of hydroxyl groups, showing the destruction of the peaks of lignocellulosic materials. The breakthrough curves revealed that for a 7.5 cm bed, adsorption performance reported the following order of promising results: Pb^2+^ > Ni^2+^ > Cd^2+^; while for a 4 cm bed, Pb^2+^ > Ni^2+^. The mechanism of adsorption of the evaluated metals onto cocoa pod husk was attributed to cationic exchange and microprecipitation due to the presence of Ca, K, and Si in the structure of the bio-adsorbent. Finally, the continuous adsorption was modeled under the mathematical expressions of Adams–Bohart, Thomas, and Yoon–Nelson reporting good fitting with correlation coefficient above 0.95.

## 1. Introduction

In recent years, water scarcity has been a risk to the maintenance of the human species, as a result of the expansion and demand for water, the destruction of the environment, industrialization, the development of population, and climate change [1]. In this sense, the discharge of industrial wastewater highly contaminated with heavy metal ions is unsafe for aquatic life due to the toxicological characteristics of these contaminant sources [2]. Studies have proven the presence of heavy metals such as copper, zinc, cadmium, and lead in rivers representing a serious problem that must be addressed by evaluating treatment techniques [3]. Lead is considered one of the most phytotoxic metals causing cell membrane damages, severe infections in digestive organs, and reproductive disorders [4,5]. This heavy metal ion mainly comes from battery and metal plating facilities [6]. Nickel also produces acute effects on organisms such as nausea, chest pain, dizziness, and cyanosis [7]. Regarding cadmium ions, adverse health effects may appear even at trace levels [8]. 

Various processes such as coagulation, ion exchange, ozone, membrane, oxidation, ionization, polyelectrolyte, and biological processes are used for treatment of contaminations in water, but practically, they have certain disadvantages such as higher cost, large amount solid waste generation, not being effective for all types of pollutants, requirement of higher land area, hazardous to human and water life, large electric demand, etc. [9]. Among the technologies for wastewater treatment, the adsorption process seems to be one of the most efficient for removal of heavy metal ions [10]. This technique is based on transferring pollutants from the effluent to a solid phase with sufficient surface to provide all active sites needed for sorbate uptake [11]. Unlike commercially available adsorbents that are expensive, several works have focused on developing adsorbents from agricultural residues such as fruit peels [12]. Some of the advantages of biosorption are low cost, high efficiency, minimization of chemical and biological sludge, no need for additional nutrients, regeneration of bio-sorbents, and the possibility of metal recovery [13]. In the adsorption of Ni^2+^, Pb^2+^, and Cd^2+^, many low cost adsorbents have been employed such as calcium alginate/spent coffee grounds composite beads [14], activated carbon from tamarind nut seed treated with phosphoric acid [15], biochars from rice husk at different conditions of pyrolysis (300, 400, 500, 600, and 700 °C) [16], natural clay [17], activated carbon prepared from Citrus limetta leaves [18], 5-sulfosalicylic acid modified lignin [19], and phosphoric acid-modified biochar generated from chicken feather [20]. 

Among them, cocoa byproducts are receiving attention as a good source of adsorptive materials owing to its availability, renewability, and low cost [21]. Cocoa (*Theobroma cacao* L.) is the fruit of cocoa tree, and its seeds are called cocoa beans. Aside from beans, the fruit is composed of a pod husk, bean shell, and pulp, representing together about 70–80% of the fruit in dry weight [22]. Despite Central and South American countries accounting for only about 14% of the world cocoa production compared to those from African countries, huge quantities of byproducts are generated during the field-processing chain [23]. Because of the environmental problems associated with final disposition of such agro-industrial residues, many researchers have proposed the use of these lignocellulosic materials for different applications such as food industry and water treatment [24].

Regarding heavy metal adsorption studies, most of the research has been done in batch systems because they are effortless to apply on a laboratory scale but challenging to use on a large scale, mainly when the volume of industrial effluent requiring treatment is large [25]. Nevertheless, data obtained during batch system essays may not apply to continuous fixed bed columns performance, because time might not be long enough to reach equilibrium [26]. Therefore, it is important to determine the practical applicability of a bio-sorbent in the continuous mode [27]. Moreover, in large-scale process operations, fixed-bed column systems are preferred because of their high effectiveness, simplicity of operation, low cost, and ability to be expanded from a laboratory process to produce higher quality effluent [9].

In this work, the continuous adsorption of heavy metal ions using packed bed columns with cocoa pod husks biomass was evaluated. The bed depth was varied from 4 cm to 7.5 cm with the aim of analyzing its effect on breakthrough curves. Adsorption modeling was applied using the mathematical expressions proposed by Adams–Bohart, Thomas, and Yoon–Nelson. Since very little information is found in the literature on bio-adsorption scaling, the objective of this work was to evaluate the effects of bed height on the removal of Pb (II), NI (II), and Cd (II) by cocoa shells in a continuous bed system.

## 2. Materials and Methods

### 2.1. Preparation of Sorbate

Each stock solution of heavy metal ions (Pb^2+^, Cd^2+^, Ni^2+^) was prepared by dissolving 0.10 g Pb(NO_3_)_2_, 0.1 g CdSO_4_, and 0.275 g NiSO_4_ (H_2_O)_6_ in deionized water to reach 100 ppm. The initial solution pH was fixed to 6 using 0.1 M caustic soda (NaOH) and hydrochloric acid solution (HCl).

### 2.2. Preparation of Adsorbent

Cocoa (*Theobroma cacao* L.) pod husks were collected from cocoa crops located in north Colombia. This agricultural residue was washed thoroughly to remove dirt particles such as tannins, resins, reducing sugars, and coloring agents [28]. Then, biomass was dried in an oven at 95 °C for 24 h in order to remove the excess moisture and prepare the material for further treatment stages [29]. The dried cocoa shells were grounded and sieved to particle size of 0.5 mm following previous works [30].

### 2.3. Chemical Composition of Biomass

The cocoa pod husks (CPH) before and after adsorption were characterized via Fourier-transform infrared spectroscopy (FT-IR) to identify functional groups most contributing to adsorption of heavy metal ions [31]. Both proximate and ultimate analysis were conducted to quantify the elemental composition of biomass (carbon, hydrogen, and nitrogen content) and the presence of ashes, cellulose, and hemicellulose. This composition was confirmed via energy dispersive spectroscopy (EDS) analysis. Table 1 summarizes the analytical methods to determine such compositional parameters. 

### 2.4. Continuos Fixed-Bed Column Study

**Figure 1:**
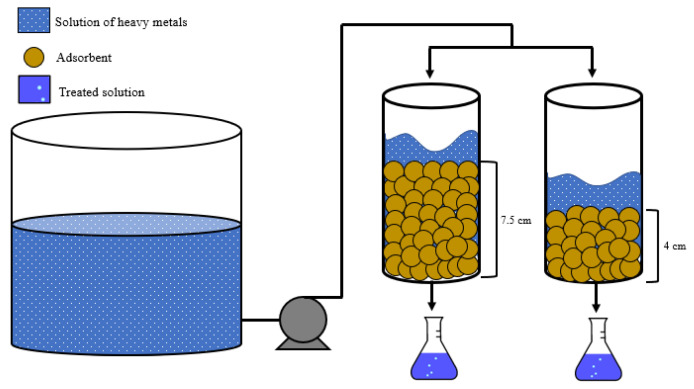
Schematic representation of experimental setup.

Figure 1 depicts the experimental set up during the continuous adsorption studies. As shown, the CPH biomass was packed in a fixed-bed column with a diameter of 6.6 cm and a peristaltic pump plug flow type was used to keep the desired flow rate of 1 mL/s. Total experiments were conducted at 25 °C, pH = 6, and initial concentration of 100 ppm. Several works related to the optimized conditions for adsorption experiments with cocoa biomasses were used to support the selection of pH and initial concentration values (see Table 2). The bed depth was varied in 4 and 7.5 cm in order to assess its effects on adsorption performance. Samples were collected from the bottom of the columns at 10, 30, 60, 90, 120, 150, 180, 210, 240, and 270 min [32]. These bed depths and time range were selected according to the results obtained in previous works related to fixed bed columns [33,34]. The remaining concentration of heavy metals (C) was measured from these samples by UV–Vis spectroscopy to evaluate the behavior of a fixed-bed column in terms of breakthrough curve, especially the breakthrough point (5% of initial concentration, i.e., C/C_o_ = 0.05; considering that the yield of the process would be 95%).

### 2.5. Adsorption Modeling 

The breakthrough curves for all heavy metal ions were built by plotting C/C_o_ as a function of time [39]. The breakthrough point (P_b_) is then identified giving the maximum loading of the biomass and the time that takes to reach this is called breakthrough time (T_b_). The adsorption capacity at breakthrough is calculated according to Equation (1), where C_o_ (mg/L) is the initial concentration of metal ions, V is the volume of the solution (L), m is the mass of adsorbent (g), and q is the amount of heavy metals ions adsorbed per weight unit of adsorbent (mg/g) [40].

(1) $$q\left(t\right)=\frac{ [C_{o}-C\left(t\right)]\;V}{m}$$

The dynamic of fixed-bed columns for adsorption of heavy metal ions is modeled by fitting experimental results of the breakthrough curve to mathematical models such as Adams–Bohart, Thomas, and Yoon–Nelson, by non-linear adjustment using the software OriginPro 2021^®^ (OriginLab, Northampton, MA, USA). 

*Adams*–*Bohart model*: This model assumes that the sorption rate is proportional to the adsorption capacity as well as the remaining concentration and is used to describe the first part of the breakthrough curve [41]. The mathematical expression is given by Equation (2), where the remaining concentration of adsorbate is function of time $C\left(t\right)$, $k_{\mathrm{AB}}$ is the kinetic constant (L/mg min), $N_{0}$ is the saturation concentration (mg/L), Z is the bed depth of the column, and F is the superficial velocity (cm/min) [42].

(2) $$\frac{C\left(t\right)}{C_{o}}=\exp\left(k_{\mathrm{AB}}C_{0}t-k_{\mathrm{AB}}N_{0}\frac{Z}{F}\right)\;\;$$

*Thomas model*: This model is based on second-order kinetics and considers that biosorption is not limited by the chemical reaction but is controlled by mass transfer at the interface [43]. The parameters of this model are determined according to the mathematical expression given by Equation (3), where $k_{\mathrm{Th}}$ is the Thomas kinetic coefficient (mL/mg min), W is the volumetric flow rate (mL/min) [42].

(3) $$\frac{C\left(t\right)}{C_{o}}=\frac{1}{1+\exp\left(k_{\mathrm{Th}}q\;m/W-k_{\mathrm{Th}}C_{0}t\right)}$$

*Yoon*–*Nelson model*: This model assumes that the rate of decrease in the probability of adsorption for each adsorbate molecule is proportional to the probability of adsorbate adsorption and the probability of adsorbate breakthrough on the adsorbent [44]. The mathematical modeling is given as follows:

(4) $$\frac{C\left(t\right)}{C_{o}-C\left(t\right)}=\exp\left(k_{\mathrm{YN}}t-{\tau k}_{\mathrm{YN}}\right)\;\;$$

where $k_{\mathrm{YN}}$ is the Yoon–Nelson rate (min^−1^) and τ is the time required for 50% adsorbate breakthrough (min) [32]. 

## 3. Results and Discussion

### 3.1. Chemical Composition of Biomass

**Figure 2:**
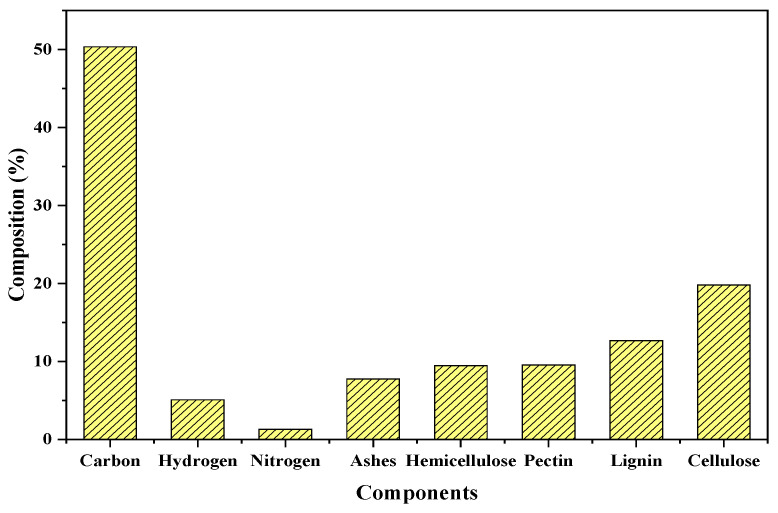
Compositional characterization of CPH biomass.

Figure 2 shows the characterization results for CPH biomass derived from both proximate and ultimate analysis. The element that most contributed to the biomass composition was carbon, followed by hydrogen and nitrogen. The ashes content was quantified in 8%, which is lower than those reported in other works. For example, Forero-Nuñez, Jochum, and Sierra [45] performed proximate analysis to cocoa pod husks and reported a composition of 13.21% for ashes. According to Lu et al. [46], the chemical composition of CPH biomass is in the range of 6.4–8.4% of ashes, 6–12.6% of pectin, 19.7–26.1% of cellulose, 4–28% lignin, and 8.7–12.8% of hemicellulose. In this work, compositions were in the above ranges supporting the high lignocellulosic content of these types of agricultural residues coming from cellulose (19%) and lignin (13%). Due to the presence of lignin, cellulose, and hemicellulose, which is why a high Pb^2+^, Cd^2+^, and Ni^2+^ adsorption efficiency is expected, because these polymers are known for the large number of hydroxyl and carboxylic groups that can favor the adsorption of heavy metals [47,48].

**Figure 3:**
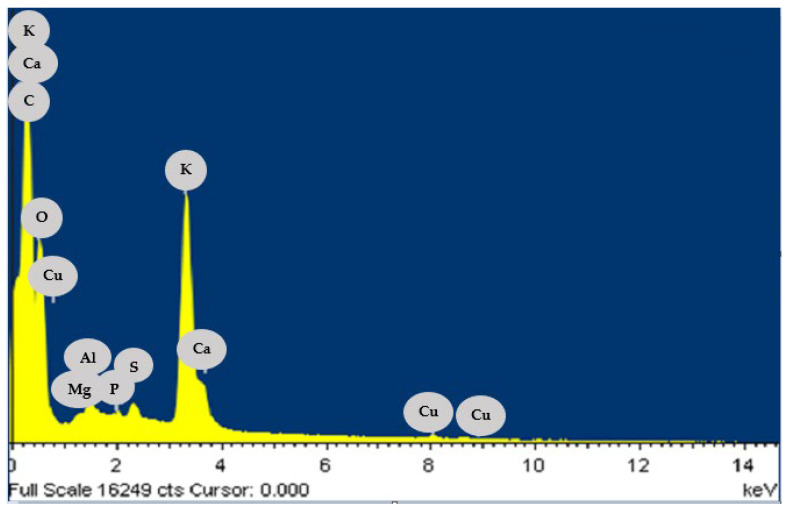
EDS spectrum of CPH biomass.

The different elementals in cocoa pod husks were confirmed from each measuring spot of EDS analysis. As shown in Figure 3, carbon, calcium, potassium, and oxygen elements reported the highest peaks in EDS spectrum. The presence of carbon and oxygen was expected due to the nature of functional groups in lignocellulosic materials [49]. Elements such as potassium and magnesium are exchangeable during adsorption by heavy metal ions and elements such as oxygen and phosphorous contribute to chelation, coordination, and complexation [50]. Similar EDS spectra were reported by Okoya, Akinyele, Ofoezie, Amuda, Olayande, and Makinde [51] for cocoa husk char. They found the following elemental composition: C (42.04%), O (37.95%), Ca (6.32%), K (4.71%), and Mg, P, Si, and Fe in lower weight percentages. It has been reported that the presence of these elements attached to the functional groups of lignocellulosic materials is associated with the ability of this type of biomasses to capture ions through ion exchange [49]. The presence of Ca, K, Mg, and Si may indicate the possibility of ion exchange process [52], considering the Pb^2+^, Cd^2+^, and Ni^2+^, may interchange with these elements on the adsorbent surface. In addition, the mechanisms of Pb^2+^, Cd^2+^, and Ni^2+^ adsorption onto biomasses can include ion exchange, micro-precipitation, complexation, and coordination owing to the presence of functional groups such as hydroxyl, carboxyl, amides, and phenols in the lignocellulosic materials [53]; however, these works identified the cation exchange mechanism and the microprecipitation mechanism as crucial in the biosorption of the heavy metals under study by the evaluated bio-sorbent [54,55].

**Figure 4:**
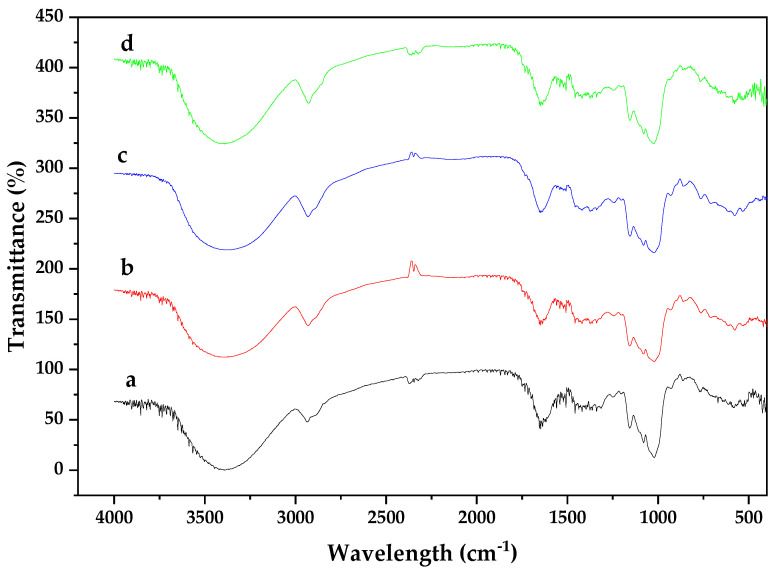
Normalized FT-IR spectra for: (**a**) CPH before adsorption, (**b**) CPH-Cd^2+^, (**c**) CPH-Ni^2+^, and (**d**) CPH-Pb^2+^.

Figure 4a depicts the FT-IR spectra for CPH biomass before and after the adsorption of heavy metal ions. This lignocellulosic material exhibited peak around 1900–2000 cm^−1^ corresponding to stretching vibrations of alkenes (C=C) [56]. Aliphatic hydrocarbons were observed at 2020 and 2190 cm^−1^ while carboxylic acid was found in a sharp adsorption band around 1550–1870 cm^−1^ [30]. The peaks at 1030 and 1150 cm^−1^ were assigned to the vibrations of thioureas and thioamides [31]. Due to the chemical composition of this biomass, the presence of carboxylic acids, phenols, and thioamides interacting in the wide spectra of the adsorbent was expected. After the adsorption process, the FT-IR spectra were recorded for the biomass loaded with all heavy metal ions. As shown in Figure 4b–d, no significant changes were observed in the spectra. The complex nature of the CPH as lignocellulosic material was proven with the presence of 71 peaks. The most representative bands in the four spectra occurred around 3200–3900 cm^−1^ that is attributed to the presence of hydroxyl groups. The peaks observed at 2928.07 and 1926.97 cm^−1^ correspond to the presence of C-H groups and the double bond groups C=C, typical of the lignin aromatic groups [57]. After the adsorption process, there was a variation in the frequency that can be attributed to the binding of the ions to the different functional groups present in the biomass, as corroborated by the change in the intensity and width of the adsorption peak of 2341 cm^−1^ due to the interaction of hydrogen bonds with overtone patterns that indicate the presence of carboxylic acids (-COOH), due to O-H stretching, as well as the change in intensity of the adsorption peak at 2927.94 cm^−1^ attributed to the vibrations of C-H methyl, methylene, and methoxy groups present in the biomass that facilitate the adsorption process.

### 3.2. Continuous Fixed-Bed Column Study

**Figure 5:**
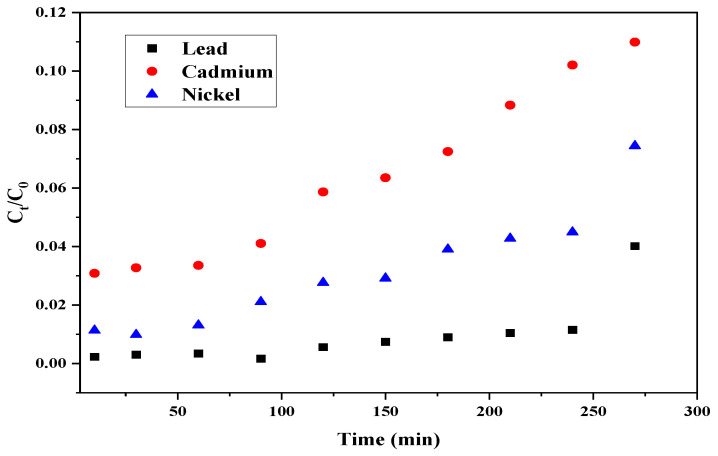
Breakthrough curves at bed depth of 7.5 cm using CPH biomass.

Figure 5 shows the breakthrough curves obtained for lead, cadmium, and nickel ions at bed depth of 7.5 cm. The time at which the breakthrough point is reached was determined for all heavy metal ions as follows: 270 min for Pb^2+^, 240 min for Ni^2+^, and 120 min for Cd^2+^ suggesting that biomass saturated faster when treating cadmium stock solutions. The main mechanisms during the removal of heavy metals using biomass-based adsorbents involve physical bonding, ion exchange, and complexation/chelation; however, the presence of carboxylic acid groups in lignocellulosic material facilitates the exchange of cation and complexation [58]. Based on this curve, the fixed-bed column with CPH biomass is more convenient to remove lead ions than cadmium ions owing to the maximum adsorption yield and breakthrough point reported in Table 3 at breakthrough times. According to Assaad et al. [59], the differences in adsorption results for a variety of heavy metals may be attributed to its ionic ions as well as hydration capacity.

**Figure 6:**
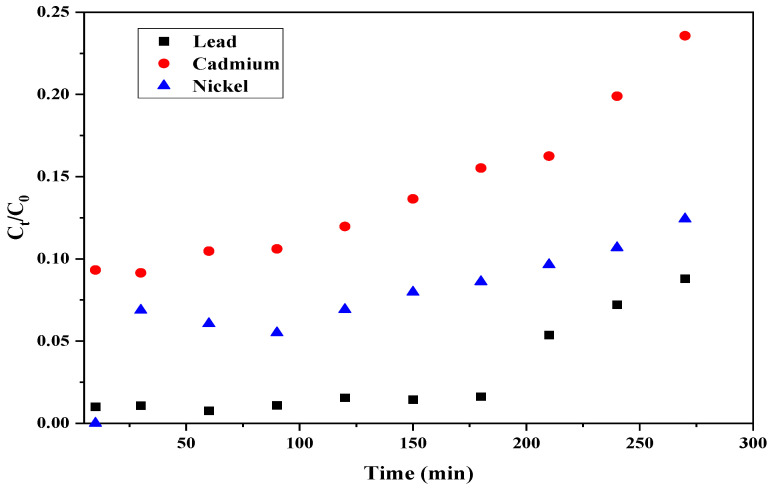
Breakthrough curves at 4 cm using CPH biomass.

As shown in Figure 6, the effluent concentration of Cd^2+^ ions reached higher concentration in a fixed-bed column at a depth of 4 cm than breakthrough concentration before the first sampling time. This result may be explained considering that the mass of adsorbent used in the 4 cm bed height column was 5 g, so it is possible that in the case of this metal, the amount of adsorption sites available are not sufficient to carry out complete adsorption. According to the removal yields for both bed depth, it can be said that 7.5 cm bed has greater efficiency than the 4 cm bed for the removal of cadmium ions. These results may be caused due to the higher amount of adsorbent increases the presence of active sites of adsorption in the adsorbent surface [60] and the residence time; therefore, the diffusion of the ions is more effective, since the contact between the phases is more intimate as reported by Khalfa et al. [61], who found that exhaustion time increased with the increase of bed depth from 2 to 6 cm. Cortés, R. [62] also evaluated the effect of two different bed depths (5 and 15 cm) for the removal of cadmium using natural zeolite. They reported a faster increase in concentration for 5 cm than for 15 cm. They estimated a breakthrough time of 1.5 and 22.5 min, respectively, which were significantly lower in comparison to those obtained using cocoa residue. In addition, when the bed height of the adsorbent was increased, binding sites were provided for the attachment of the heavy metal molecules for adsorption, and, with the bed height increased, effluent volume is increased that due to the more contact time [61]. Moreover, it is observed that the slope of the breakthrough curve was reduced based on the increasing bed height due to the expansion of the mass-transfer zone [63].

For lead ions, it was found that increasing the depth of the packed column also increased the breakthrough time by 60 min. Dávila-Guzmán, N. [64] conducted the assessment of lead removal in a fixed bed column filled with coffee husks and used different bed depths (7, 14, and 21 cm). They found that breakthrough time was 80, 220, and 500 min, respectively, which confirmed the direct influence of column design on adsorption results. For nickel ions, the breakthrough time varied from 60 to 240 min when increasing bed depth, similar to the results previously reported by Tejada-Tovar, Villabona-Ortíz, Alvarez-Bajaire, Jattin-Torres, and Acevedo [30]. Based on the maximum removal yields and breakthrough point, lead adsorption was more promising than nickel and cadmium under this evaluated continuous system. 

For both evaluated bed heights, it was corroborated how adsorption occurs in continuous systems, where the greatest removal of the metal ion occurs in the first moments of contact with the adsorbent biomass [65]. As summarized in Table 3, breakthrough time increases as bed depth increases for all heavy metal ions. For cadmium ions and 4 cm bed, it was not possible to calculate the adsorption capacity Q_b_; this may be due to the insufficient contact time between the biomass filling the column and the adsorbate, causing the diminution of the amount of adsorbed heavy metal [66]. For the 7.5 cm bed, adsorption performance reported the following order Pb^2+^ > Ni^2+^ > Cd^2+^; while for the 4 cm bed, Pb^2+^ > Ni^2+^. This affinity of the active sites in the adsorbent for the Pb^2+^ (7.18) ions may be due its higher covalent index than Ni^2+^ (5.73) and Cd^2+^ (5.51) [67], as well as their ionic binding indexes based on their electronegativity 2.33, 1.91, and 1.61 for Pb^2+^, Ni^2+^, and Cd^2+^, respectively [68]. According to the classification criteria above, higher values indicate stronger bonds. It has been reported that, relative to ionic interactions, metal ions exhibiting high covalent indices possess high and effective chelating affinity towards ligands. Due to the high covalent index of lead ions, they are more likely to form covalent bonds with the ligands of the adsorbent (cocoa husk) than nickel and cadmium ions, which enhances their adsorption capacity [67]. From the behavior of the breakthrough curves, it can be said that the very low slope of the curve is due to the low concentration difference between the absorbent solution and the solute of the dissolution after a few minutes of initiated the process, creating a very low concentration gradient, resulting in a weak driving force responsible for the transport of the contaminant from the fluid to the particle structure, which decreases the diffusion coefficient, causing the saturation time of the biomaterial to be increased [66].

### 3.3. Adsorption Breaktrough Curves Modeling 

**Figure 7:**
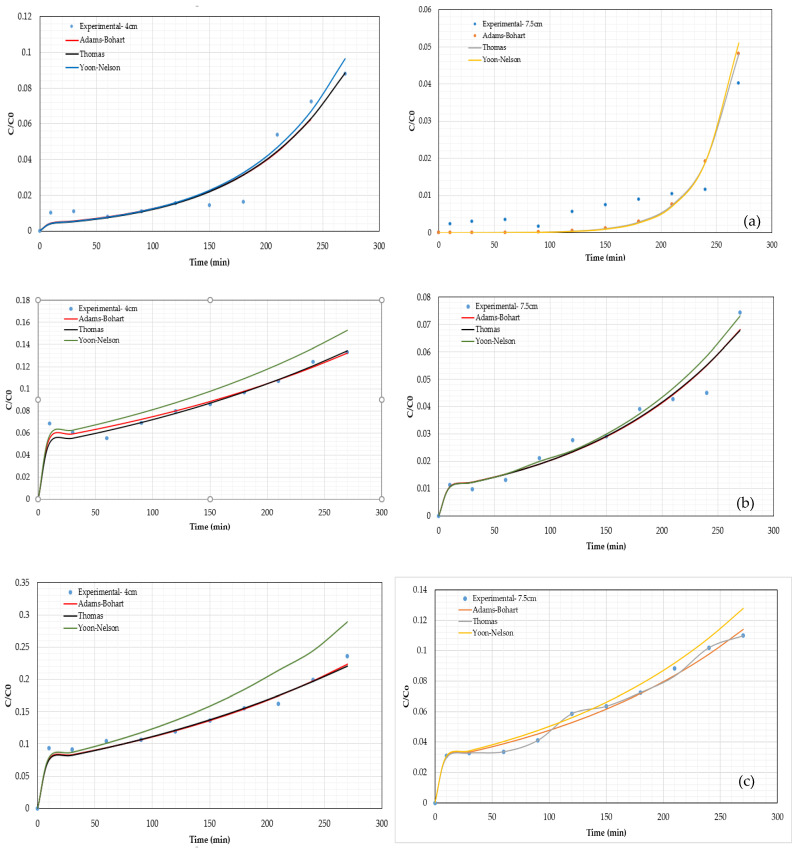
Breakthrough curves modeling at 4 and 7.5 cm for (**a**) Pb^2+^, (**b**) Ni^2+^ and (**c**) Cd^2+^.

The experimental data were employed to determine parameters of models (Adams–Bohart, Thomas, and Yoon–Nelson) that are widely used to predict adsorption performance in a packed bed column as shown in Figure 7 for each bed height evaluated. 

Table 4 shows the values for all parameters considered in the mathematical expressions (Equations (2)–(4)). A comparison of predicted models’ curve and experimental data is provided by the correlation coefficient R^2^. It was found that all models considered in this work fitted breakthrough curves with R^2^ above 0.95. The best-fitting results were observed for cadmium ions using a 7.5 cm bed because of the R^2^ = 0.99.

## 4. Conclusions

This work attempted to study the removal of heavy metal ions (Pb^2+^, Cd^2+^, Ni^2+^) from aqueous solution using a packed bed column filled with cocoa (*Theobroma cacao* L.) pod husks, which were used as adsorbent. The chemical composition of biomass showed a high contribution of carbon, followed by hydrogen. The content of lignin and cellulose confirms the characteristics of these types of materials. For the FT-IR analysis of biomass before and after adsorption, the most representative bands occur around 3200–3900 cm^−1^ attributed to the presence of hydroxyl groups. The breakthrough curves were constructed from the experimental data and revealed that for the 7.5 cm bed, adsorption performance reported the following order of promising results: Pb^2+^ > Ni^2+^ > Cd^2+^; while for the 4 cm bed, Pb^2+^ > Ni^2+^. Finally, the continuous adsorption was modeled under the mathematical expressions of Adams–Bohart, Thomas, and Yoon–Nelson reporting good fitting with correlation coefficient above 0.95.

## Figures and Tables

**Table 1:** Analytical methods for chemical characterization of biomass.

Parameter	Method
Carbon (%)	AOAC 949.14
Hydrogen (%)	AOAC 949.14
Nitrogen (%)	Total Kjeldahl nitrogen
Ashes (%)	Thermogravimetry
Pectin (%)	Digestion-thermogravimetry
Lignin (%)	Photocalorimetry
Cellulose (%)	Digestion-thermogravimetry
Hemicellulose (%)	Digestion-thermogravimetry
Elemental composition	EDS analysis
Functional groups	FT-IR analysis
Carbon (%)	AOAC 949.14

**Table 2:** Optimum conditions of pH and initial concentration reported in recent contributions for heavy metals uptake using adsorbents derived from cocoa residues.

Heavy Metal Ion	Optimum Operating Conditions	Reference
Ni^2+^, Co^2+^	C_o_ = 60–100 ppm	[35]
Pb^2+^, Cu^2+^	pH = 6	[36]
Ni^2+^	C_o_ = 25–150 ppm	[37]
Hg^2+^	pH = 6–8	[38]
Ni^2+^	pH = 6	[37]
Ni^2+^, Co^2+^	C_o_ = 60–100 ppm	[35]

**Table 3:** Breakthrough point for heavy metal ions.

Metal	Bed Depth (cm)	T_b_ (min)	Q_b_ (mg/g)	Maximum Removal Yield (%)
Pb^2+^	4	210	18	98.99
	7.5	270	25.2	99.76
Ni^2+^	4	30	6.98	93.27
	7.5	240	14.31	98.80
Cd^2+^	4	-	-	90.60
	7.5	120	7.2	96.90

**Table 4:** Parameters for continuous adsorption modeling.

Model	Parameter	Pb^2+^		Ni^2+^		Cd^2+^	
		7.5 cm	4 cm	7.5 cm	4 cm	7.5 cm	4 cm
Adams–Bohart	K_AB_ (L mg^−1^ min^−1^)	2.01 × 10^−4^	1.15 × 10^−4^	7.48 × 10^−5^	3.73 × 10^−5^	5.13 × 10^−5^	4.13 × 10^−5^
	N_0_ (mg L^−1^)	1903.88	1121.24	1561.73	3837.16	1621.07	2775.80
	SE	2.08 × 10^−5^	5.79 × 10^−5^	1.88 × 10^−5^	3.31 × 10^−5^	1.20 × 10^−5^	8.43 × 10^−5^
	R^2^	0.95	0.96	0.97	0.97	0.99	0.98
Thomas	K_TH_ (mL mg^−1^ min^−1^)	0.207	0.122	0.079	0.043	0.055	0.048
	q_0_ (mg g^−1^)	25.66	55.36	38.01	89.34	38.52	64.03
	SS	2.07 × 10^−5^	5.75 × 10^−5^	1.92 × 10^−5^	4.14 × 10^−5^	1.11 × 10^−5^	1.05 × 10^−4^
	R^2^	0.95	0.96	0.97	0.97	0.99	0.98
Yoon–Nelson	K_YN_ (mL mg^−1^ min^−1^)	0.021	0.012	7.57 × 10^−3^	3.82 × 10^−3^	5.49 × 10^−3^	5.02×10^3^
	SE	2.19 × 10^−5^	6.62 × 10^−5^	2.35 × 10^−5^	4.57 × 10^−5^	1.45 × 10^−5^	1.95 × 10^−4^
	R^2^	0.95	0.96	0.97	0.97	0.98	0.99

Where SE: statistical error.

## Data Availability

The data that support the results of this study are available on request from the corresponding author.
